# Reducing waiting time and raising outpatient satisfaction in a Chinese public tertiary general hospital-an interrupted time series study

**DOI:** 10.1186/s12889-017-4667-z

**Published:** 2017-08-22

**Authors:** Jing Sun, Qian Lin, Pengyu Zhao, Qiongyao Zhang, Kai Xu, Huiying Chen, Cecile Jia Hu, Mark Stuntz, Hong Li, Yuanli Liu

**Affiliations:** 1 School of Public Health, Chinese Academy of Medical Sciences & Peking Union Medical College, No.5 Dongdansantiao, 100730 Beijing, People’s Republic of China; 2 Fujian Provincial Hospital, 134 East Street, 350001 Fuzhou, Fujian Province People’s Republic of China; 3 School of Nursing, Shengli Clinical Medical Hospital, Fujian Medical University, 134 East Street, 350001 Fuzhou, Fujian Province People’s Republic of China; 4 Deerfield Institute, K Wah Center 3703, Middle Huaihai Road 1010, 200031 Shanghai, People’s Republic of China; 5 Deerfield Institute, 780 Third Avenue, 37th Floor, New York, NY USA

**Keywords:** Waiting time, Outpatient, Satisfaction, Interrupted time series

## Abstract

**Background:**

It is globally agreed that a well-designed health system deliver timely and convenient access to health services for all patients. Many interventions aiming to reduce waiting times have been implemented in Chinese public tertiary hospitals to improve patients’ satisfaction. However, few were well-documented, and the effects were rarely measured with robust methods.

**Methods:**

We conducted a longitudinal study of the length of waiting times in a public tertiary hospital in Southern China which developed comprehensive data collection systems. Around an average of 60,000 outpatients and 70,000 prescribed outpatients per month were targeted for the study during Oct 2014-February 2017. We analyzed longitudinal time series data using a segmented linear regression model to assess changes in levels and trends of waiting times before and after the introduction of waiting time reduction interventions. Pearson correlation analysis was conducted to indicate the strength of association between waiting times and patient satisfactions. The statistical significance level was set at 0**.**05.

**Results:**

The monthly average length of waiting time decreased 3**.**49 min (*P* = 0**.**003) for consultations and 8**.**70 min (*P* = 0**.**02) for filling prescriptions in the corresponding month when respective interventions were introduced. The trend shifted from baseline slight increasing to afterwards significant decreasing for filling prescriptions (*P* =0.003). There was a significant negative correlation between waiting time of filling prescriptions and outpatient satisfaction towards pharmacy services (*r* = −0**.**71, *P* = 0**.**004).

**Conclusions:**

The interventions aimed at reducing waiting time and raising patient satisfaction in Fujian Provincial Hospital are effective. A long-lasting reduction effect on waiting time for filling prescriptions was observed because of carefully designed continuous efforts, rather than a one-time campaign, and with appropriate incentives implemented by a taskforce authorized by the hospital managers. This case provides a model of carrying out continuous quality improvement and optimizing management process with the support of relevant evidence.

## Background

Patient waiting time for healthcare services is identified by the World Health Organization (WHO) as one of the key measurements of a responsive health system. Patient waiting time is the amount of time for patients seeking care at healthcare units before being attended for consultation and treatment [1, 2]. The United States (US) Institute of Medicine’s report “Crossing the Quality Chasm” outlines a framework of six guiding principles to staying ahead in a more competitive healthcare delivery system. One of these principles is the ability to provide timely care and to reduce harmful delays [3]. The Patient’s Charter of the United Kingdom (UK) Government sets a series of standards which state that all patients must be seen within 30 min of their appointment time [4]. It is globally agreed that a well-designed healthcare service management system should not have patients to wait long time for appointment and consultation.

Lengthy waiting time has long been considered frustrating to patients and thus appears to be a consistent and significant potential cause of patient dissatisfaction. A strong inverse relationship between patient satisfaction and waiting time has been demonstrated by many studies [5–10]. As healthcare solutions become more personalized and consumer-driven, the need to provide overall patient satisfaction is becoming more important [11].

The outstanding waiting time problem in the Chinese health systems lies in two aspects, one is long waiting time at the registration and admission windows. This is mainly due to that most Chinese hospitals used not to schedule the appointment, patients get registered upon arrival to hospitals at the service window, thus the unplanned patient flow clogged in hospitals. A straightforward and easy appointment-scheduling process is the first step for patients’ timely access to healthcare services. Multiple appointment-scheduling methods (including web-, landline-, smart phone-, and automatic teller machine “ATM”-based methods) have been used to replace the traditional process where patients were asked to make the appointment physically inside hospitals [12–14].

The other aspect of the problem is the long waiting time between the appointment time and the time patients are attended by doctors. Findings from the 2015 China National Patient Survey [15] from 136 public tertiary hospitals showed that outpatient users of ambulatory services were least satisfied with long waiting times for consultations. The key reason behind this is that the increasing of patient demand is faster than that of the health care resources. Although the number of public tertiary hospitals in China doubled during the past 10 years (from 946 in 2005 to 1972 in 2015), the annual number of outpatient visits increased nearly four times (from 397 million to 1.5 billion) during the same time period [16]. The increasing patient turnover may have implications for the overall quality of care, such as insufficiencies in patient safety and increased tensions between doctors and patients [17–19]. Therefore, the rapidly increasing demand and limited health care resources require health services must rely on improved flow control and better capacity allocation to minimize the negative effect of patient long waiting time. Organizational and structural changes must be introduced with purposeful planning and demand-oriented scheduling of outpatient care.

This study focus on the waiting times for consultations and filling prescriptions (Chinese health care system does not separate prescription from dispensing, hospitals run their own pharmacies to serve outpatients, and majority of outpatients fill prescriptions from the hospital pharmacies).

Many interventions aiming to reduce waiting times for consultation, tests and filling prescriptions have been implemented in the Chinese public tertiary hospitals [20–22]. Few interventions were well-documented and intervention impact was rarely measured with robust method. This study takes one provincial hospital as a typical example of a Chinese public tertiary hospital in southern China. It is on the top of the three-tiered public health service delivery system (community health center/station and township health center/village clinics as primary level, city secondary hospitals and county hospitals as the secondary level, and tertiary hospitals mainly located in urban areas), and public facilities dominate the overall health service delivery systems. We comprehensively document the studied hospital’s efforts to achieve the organizational and structural changes in order to reduce waiting times for consultations and filling prescriptions. The study also used longitudinal data series before and after the intervention which deliver robust results, quantitatively presents the amount that waiting time was reduced, and to what extent such efforts helped to improve the outpatient satisfaction.

## Efforts to reduce waiting times and to improve outpatient satisfactions in the studied hospital

### Baseline investigation and bottlenecks identification

An interdisciplinary taskforce was setup and authorized by the hospital manager to conduct the baseline investigations aimed to identify the deficits. The taskforce observed the performances of each outpatient doctor of the key clinical departments (the most busy and crowded departments) for one full outpatient unit time (a half working day, either between 8 and 12 am or 1-5 pm) per doctor, monitored the logging on times to their working computers (on-duty starting time) and time intervals between consultations, identified their attended patients, and extracted the waiting times of the targeted patients from the hospital health information system. The investigation was completed during 27 July-10 October 2015. Four months later, the taskforce also conducted baseline investigation at the outpatient pharmacy, which extracted the waiting times of all prescribed outpatients during 22–26 February 2016, measured the length of times of prescribed outpatients waiting for filling prescriptions from 7:30 in the morning to 6:30 in the afternoon..A total of 10,868 ambulatory patients were targeted for the baseline investigation for consultation, their average waiting time was 57 ± 30 min and the waiting time in the morning was longer than that in the afternoon. Key departments which had longer waiting time than the average were identified. The average length of consultation time was about five minutes. The total number of targeted prescribed patients were 17,235, their daily average waiting time for filling prescription was 34 ± 19 min, and waiting time was the longest (>40 min) during 10:30–14:00.

### Root cause analysis

The taskforce identified the key factors affecting efficient segregation of patients, which include late-arrival and early leave of physicians; more visiting patients in the morning than in the afternoon; more patients and follow-up patients as well as less on-duty doctors in some specific departments; shorter time interval between consultations (four minutes) than the length of consultation times of some physicians; poor scheduling of receiving patients; and late arrival of appointed patients. For the pharmacy service, the critical deficit was short of on-duty staff, especially in the period of 12:00–14:30 and after 17:30.

### Comprehensive interventions

The taskforce formulated a series of interventions aimed to reduce waiting time for consultations, which include 1) procedure changes: simplifying the appointment scheduling, assigning patients with more accurate estimation of time interval for consultation based on the baseline investigation (five minutes), allocating separated consultation rooms to each on-duty doctor, setting up a help desk to re-schedule the late arrived appointed patients and patients who need help; 2) supply side changes: checking on the attendance of on-duty doctors and giving financial penalties to late arrivals and early leaves, informing on-duty doctors in advance about the number of appointments and on-time reminders through sending text message to the mobile phones on the eve of the on-duty day, and when the on-duty doctors being late for more than 10 min; naming and shaming the bad performances at weekly regular meetings; 3) demand side changes: informing and reminding visiting patients the time of consultations through sending text messages via Application (APP) set in the smart phone; and strengthening appointment scheduling through various approaches, patients who are late for more than 15 min will have to make new appointment.

In terms of the interventions to reduce waiting times for filling prescriptions, the taskforce increased the number of on-duty staff and windows during peak hours; strengthened on-duty staff in the period of 12:00–14:30 and after 17:30; informed patients to be prepared for filling prescriptions through showing electronic card number of patients on the light emitting diode (LED) screen outside the pharmacy; and informed patients with the number of patients on the waiting list through sending text message via APPs set in the smart phone.

## Methods

### Study design

We conducted a longitudinal study about the length of waiting times for consultations between 1 October 2014 and 28 February 2017, and for filling prescriptions between 1 March 2015 and 28 February 2017 in the studied hospital with a quasi-experimental design of before and after assessments.

### Population and setting

The study populations related to waiting times for consultations consisted of all visiting outpatients of the studied hospital during 1 October 2014–28 February 2017 (excluding the easily accessible outpatients who only requested repeat prescriptions), while the study populations related to filling prescriptions included all patients who filled prescriptions from the outpatient pharmacy during 1 March 2015–28 February 2017. The studied hospital was a public provincial tertiary general hospital located in the capital city of Fujian Province, where there are approximately 2500 daily outpatient visits and 2800 prescriptions filled.

### Data sources

#### Routinely collected data extracted from the hospital information system

We obtained all relevant information (including process management, personal, and clinical information) from different information systems. Each visiting outpatient is given an electronic patient card with a unique identity code at the registration desk. The electronic patient card can be used at any service point within the hospital, including the appointment system, the registration system, working computers of doctors, nurses, labs, pharmacists, and payment. All process management, diagnosis, and treatment information are recorded in the electronic patient card. Waiting times for consultations and filling prescriptions were calculated based on the time points of registration, consultation, prescription bill payment, and dispensing. These times are recorded in different management models as soon as the electronic patient card is swiped at respective computer working stations. The sequence numbers and exact times for consultation and dispensing are automatically allocated by the hospital information system to each visiting outpatient with a printed receipt upon registration and completing payment of prescription bill.

#### Data obtained from the patient satisfaction survey

Upon approval of the Hospital Ethics Committee, starting from 1 January 2016, the taskforce started to conduct daily patient satisfaction survey, who invited outpatients to fill approximately 50 questionnaires every day. The structured Likert five-point scale questionnaire was pre-installed into i-pad. An Informed Consent statement was read by the taskforce staff before filling the questionnaire, and only the patients who have no objections responded to the survey. One specific indicator of the questionnaire used by the studied hospital is about pharmacy services (“Are you satisfied with pharmacy services?”), and another is about the consulting doctor (“Are you satisfied with the consulting doctor?”). Patients were asked to rate their satisfaction to each indicator. The outpatient respondents were identified from the waiting area outside the outpatient pharmacy during working hours every day using convenient sampling method. A total of around 1000 responded outpatients constitute the sample size of monthly average outpatient satisfaction score calculations for respective healthcare service after excluding the non-experienced patients. Patient satisfaction results were used as a tool to evaluate the performance of on-duty doctors and pharmacists, and financial penalties were given to poor performance. All these were integrated into the routine management.

### Outcome measures

We defined the length of waiting time for consultation as the time period between the moment when the consultation is automatically allocated to each visiting outpatient by the hospital information system upon their registration, and the moment when the electronic patient card is recorded by the doctor in the computer system and the patient is attended by the on-duty doctor. We also defined the length of waiting time for filling prescriptions as the time period between the moment when the prescription bill is paid (the electronic patient card is swiped at the payment ATM machine or the payment window and a receipt is printed out) and the exact time when the patient’s name and sequence number are shown on the LED screen outside the outpatient pharmacy. The following indicators were employed to measure waiting times and patient satisfactions:

#### The monthly average length of waiting time for consultations (the studied hospital data)

Measured by having the exact time when the electronic patient card is recorded by the doctor in the computer system, minus the exact time of appointment time. The averages of the above results of all visiting outpatients in each month during 1 October 2014–28 February 2017 were calculated.

#### The monthly average length of waiting time for filling prescriptions (the studied hospital data)

Measured by having the exact time when the sequence number and the name of the prescribed outpatient are shown on the LED screen outside the outpatient pharmacy, minus the exact time when the payment of the prescription bill is completed. The averages of the results of all prescribed outpatients in each month during 1 March 2015–28 February 2017 were calculated.

#### The monthly average outpatient satisfaction scores towards consulting doctors and pharmacy services (the survey conducted by the studied hospital)

Measured by having 5, 4, 3, 2, and 1 assigned to each Likert scale respectively, having the sum of scores of “very satisfied” and “satisfied” divided by the sum of the scores of all five scales (“very satisfied”, “satisfied”, “neither satisfied nor unsatisfied”, “unsatisfied” and “very unsatisfied”), and multiplying by 20 for each respective indicator to obtain the centesimal satisfaction score.

### Statistical analysis

We used monthly average length of waiting time data and corresponding outpatient satisfaction data to assess trajectories in waiting times for consultation and filling prescriptions, as well as outpatient satisfactions towards consulting doctors and pharmacy services over time. We analyzed the time series data using a segmented linear regression model with statistical software (SPSS 21.0) to assess changes in levels and trends of waiting times for consultations and for filling prescriptions before and after the introduction of respective waiting time reduction interventions. Interrupted time series analysis statistical software can control for auto-correlated errors, and can also adjust for potential serial correlation of the data [23, 24]. We regarded September 2015 and February 2016 as the intervention time points for reducing waiting times for consultations and for filling prescriptions, respectively. Segmented linear regression divides the time series into pre- and post-September 2015 and pre- and post-February 2016 segments. We also compared the changes in trends and levels of waiting times before and after implementation of the respective waiting time reduction interventions. Regression analysis was also conducted for the outpatient satisfaction scores towards the consulting doctor and towards pharmacy services. Pearson correlation analysis was conducted to indicate the strength of association between waiting times and respective patient satisfactions. The statistical significance level was set at 0.05.

## Results

The monthly average waiting times for consultations and for filling prescriptions before and after the respective interventions are presented in Appendix 1. The waiting times for consultations ranged between 20.88–23.92 min during October 2014–August 2015, which reduced to a range between 15.83–20.32 min during September 2015–February 2017; and the waiting times for filling prescriptions ranged between 24.91–42.52 min during March 2015–January 2016, which went down to a range of 14.99–28.77 min during February 2016–February 2017. Appendix 2 shows the monthly average satisfaction scores towards consulting doctors and pharmacy services. From February 2016 to February 2017, the monthly average satisfaction scores towards consulting doctors ranged from the lowest of 82.78 to the highest of 93.44; which for filling prescriptions ranged from the lowest of 59.33 to the highest of 89.52.

**Fig. 1:**
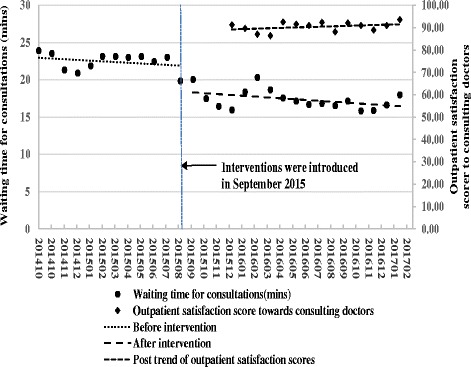
Level and trend changes of waiting times for consultations and the outpatient satisfaction scores towards consulting doctors

**Fig. 2:**
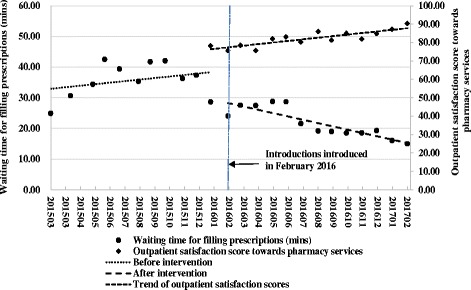
Level and trend changes of waiting times for filling prescriptions and the outpatient satisfaction scores towards pharmacy services

As presented in Table 1 and Figs. 1 and 2, the segmented regression analysis results indicate that, prior to the interventions on waiting time for consultations in September 2015, the trend of the monthly average length of waiting time for consultations declined slightly (*P* = 0.37). In September 2015, the month that consultation interventions were introduced, there was an immediate decrease in average length of waiting time for consultations of 3.49 min (95% CI: -5.38 – -1.61; *P* = 0.003). This decreasing trend continued after September 2015 through the end of the study period in February 2017, although the results were not statistically significant (*P* = 0**.**07).

**Table 1:** Estimated level and trend changes of waiting times and outpatient satisfaction scores before and after the respective interventions

Outcome variable	Coefficient		95% CI	*P*-value
Waiting time for consultation	Intercept	23.05	/	/
	Baseline trend	−0.09	−0.31 ~ 0.12	0.37
	Level change	**−3.49**	−5.38 ~ −1.61	**0.003**
	Trend change	−0.12	−0.24 ~ −0.03	0.07
Waiting time for filling prescription	Intercept	32.88 0.479	/	/
	Baseline trend		−0.39 ~ 1.35	0.26
	Level change	**−8.70** **−1.09**	−16.12 ~ −1.29	**0.02**
	Trend change		−1.76 ~ −0.42	**0.003**
Outpatient satisfaction score towards consulting doctors	Intercept	4.41	/	/
	Trend change	0.01	−0.005 ~ 0.03	0.17
Outpatient satisfaction score towards pharmacy services	Intercept	3.91	/	/
	Trend change	**0.05**	0.03 ~ 0.07	**0.000**

Bold signifies statistically significant coefficient (*P* < 0.05)

For the waiting time of filling prescriptions, the trend steadily increased (*P* = 0**.**26) before the interventions in February 2016. There was an immediate decrease in average length of waiting time of 8**.**70 min (95% CI: -16**.**12 – -1.29; *P* = 0**.**02) in February 2016, when the interventions were introduced. There was a significant decreasing trend in average waiting time from February 2016 through the end of the study period in February 2017 (*P* = 0**.**003).

The regression results of respective monthly average outpatient satisfaction scores during January 2016 – February 2017 indicate that satisfaction towards consulting doctors slightly increased (*P* = 0**.**17), and satisfaction towards filling prescriptions significantly increased (*P* < 0**.**05).

The Pearson correlation analysis results indicate that the strength of negative correlation between the waiting time of filling prescriptions and outpatient satisfaction score towards pharmacy services (*r* = −0**.**71, *P* = 0**.**004) is stronger than that between the waiting time of consultations and outpatient satisfaction score towards consulting doctor (*r* = −0**.**39, *P* = 0**.**17).

## Discussion

Huang [25] found that outpatients were reasonably satisfied if they waited no more than 37 min when arriving on time. Our results are in line with this finding. The waiting time for consultations in the studied hospital did not exceed this threshold, though the waiting times for filling prescriptions far exceeded this limit, before interventions were introduced. Outpatients were less satisfied with pharmacy services. There were negative correlations between waiting times and outpatient satisfaction scores. Such correlations between waiting time for filling prescriptions and outpatient satisfaction score towards pharmacy services was stronger than that between waiting time for consultations and outpatient satisfaction score towards consulting doctors. It is likely that patients see the filling of a prescription as more of process and therefore strongly influenced by time, whereas there are different expectations of the doctor consultation.

The regression analysis shows the effectiveness of the interventions on reducing waiting times for consultation and for filling prescriptions in the studied hospital. There were statistically significant immediate intervention effects on reduction of waiting time for consultations. Statistically significant long term reduction effects on waiting time for consultations was also shown, implying that waiting time reduction interventions in the studied hospital were not a one-time campaign but continuous efforts. Such continuous efforts are essential for quality of healthcare improvements. Findings provide evidence in support of the effectiveness of the interventions. By the end of February 2017, all the waiting times were below the threshold set by the Patient’s Charter of the UK Government.

Many studies proved that, process matters in healthcare, a process improvement team approach for evaluating and redesigning the patient care system can be successful in reducing waiting times and raising patient satisfactions [26–28]. Similar findings were also concluded by many studies in the Chinese tertiary hospitals [29–31]. As an example of Chinese tertiary public hospitals, who has been trying to address the waiting time problem, the studied hospital provides a good example of carrying out continuous quality of healthcare improvements in optimizing the process management in outpatient care with the support of relevant evidence. Firstly, a multidisciplinary taskforce empowered by the top hospital manager with supreme authority to design interventions, to appraise the performance of the targeted players, and to link the appraisal results with financial penalties, such process-improvement approach with major sustained support from top-level hospital administrators is fundamental for quality improvement. Secondly, they selected the problem of waiting times and clearly defined it. This enabled the interventions to be carried out with clear objectives and targets. Thirdly, they identified the potential cause of the problem, collected and analyzed data related to the problem, generated solutions to address the root causes of the problem, and gained support from the top hospital manager to secure the credibility. Fourthly, they implemented the solutions on a trial of waiting time for consultations, and expanded to waiting time for filling prescriptions, which secured steady push forward of the interventions step by step. Fifthly, they evaluated the results and gathered and analyzed the data on the solutions, which made evidence based solution adjustment possible. Finally, the integrated information system was shown to be indispensable for implementing the above problem solving process efficiently.

It is a pity that the outpatient satisfaction survey started after the interventions on waiting time for consultations, and just one month before the introduction of the interventions on waiting time for filling prescriptions. We were not able to measure the changes of the outpatient satisfaction scores corresponding to the changes of the waiting times for both consultations and filling prescriptions. The upward trends of the monthly average outpatient satisfaction scores towards both consultations and filling prescriptions already support the effective effects of interventions on waiting times, and the positive impacts of waiting time reductions on raising the outpatient satisfaction scores. However, as literatures also proved that time spent with the physician is a stronger predictor of patient satisfaction than is the time spent in the waiting room [32], the increased patient satisfaction might not be brought by shorter waiting time but longer consulting time due to process improvement. The daily outpatient satisfaction survey carried out by the studied hospital only asks the outpatients’ satisfactions with consulting doctors instead of waiting time for consultations. This is also the case for the outpatients’ satisfaction with pharmacy services, which does not specifically target patients’ satisfaction towards waiting time for filling prescriptions. Although waiting time is an important factor influencing the outpatient satisfaction, there are still many other critical factors affecting the outpatient satisfaction with consulting doctors. Previous studies have found that interpersonal relationships are very important in patient satisfaction, and therefore patients who had to wait but then had a good experience with their doctor are still likely to be satisfied [33]. Ideally we would be able to analyze both the waiting time and the outpatient satisfaction time series data with the segmented regression analysis, which would generate strong evidence of correlation between waiting time reduction and outpatient satisfaction improvement, as a further proof of the Pearson correlation analysis. However, due to the absence of the outpatient satisfaction data before 2016 (the hospital outpatient satisfaction survey started in January 2016), we can only show the trend of outpatient satisfaction after interventions.

## Conclusions

The evidence generated by robust method proved that the studied hospital used its integrated health information system to support a well-designed and carefully arranged quality improvement in reducing waiting time and raising patient satisfaction. This typical case set up an example for the other Chinese public tertiary hospitals, as well as the overloaded public hospitals in other settings to implement organizational and structural changes in order to address the waiting time issue.

## Appendix 1

**Table 2:** Waiting times for consultations and for filling prescriptions of the studied hospital

Month	Number of outpatients (excluding outpatients who filled repeat prescriptions)	Length of waiting times for consultations (mins) mean ± SD	Number of outpatients who filled prescriptions	Length of waiting times for filling prescriptions (mins) mean ± SD
201410	66,336	**23.93 ± 26.22**	/	/
201411	65,707	23.54 ± 26.23	/	/
201412	69,923	21.32 ± 24.84	/	/
201501	65,183	20.88 ± 24.79	/	/
201502	48,111	21.88 ± 25.37	/	/
201503	68,158	23.15 ± 26.46	84,616	24.91 ± 19.09
201504	68,526	23.14 ± 26.30	85,034	30.66 ± 23.61
201505	64,930	23.01 ± 25.94	78,996	34.41 ± 24.94
201506	65,657	23.10 ± 25.93	78,531	**42.52 ± 31.35**
201507	68,988	22.44 ± 25.53	81,879	39.36 ± 28.26
201508	63,395	23.03 ± 26.04	77,129	35.34 ± 27.31
201509	62,383	19.86 ± 24.65	75,583	41.76 ± 31.21
201510	65,381	20.06 ± 24.85	73,338	42.08 ± 31.50
201511	68,748	17.48 ± 22.50	75,693	36.30 ± 24.72
201512	73,806	16.44 ± 21.81	82,285	37.35 ± 25.64
201601	64,189	15.96 ± 21.66	68,918	28.66 ± 22.40
201602	51,074	18.37 ± 22.89	37,168	24.05 ± 14.15
201603	74,871	20.32 ± 24.61	78,711	27.59 ± 14.80
201604	70,623	18.67 ± 23.49	74,570	27.46 ± 14.94
201605	74,313	17.57 ± 22.55	78,181	28.77 ± 15.87
201606	69,915	17.14 ± 22.26	75,317	28.68 ± 18.11
201607	72,726	16.69 ± 22.26	75,574	21.61 ± 13.63
201608	77,617	16.84 ± 21.99	81,185	19.20 ± 12.55
201609	68,099	16.53 ± 22.07	76,600	18.97 ± 12.71
201610	66,473	17.15 ± 22.79	73,528	18.53 ± 12.46
201611	74,222	**15.83 ± 20.95**	80,994	18.57 ± 12.05
201612	75,590	15.88 ± 21.39	86,109	19.31 ± 12.18
201701	58,151	16.62 ± 21.48	66,611	16.04 ± 11.16
201702	62,359	18.00 ± 22.65	66,424	**14.99 ± 10.67**

1. Bold signifies maximum and minimum results before and after the implementation of respective interventions

## Appendix 2

**Table 3:** Results of the monthly outpatient satisfaction survey toward consulting doctors and pharmacy services conducted by the studied hospital

	Outpatient satisfaction towards consulting doctors						Outpatient satisfaction towards filling prescriptions					
Time	Very satisfied N (%)	Satisfied N (%)	Neither satisfied nor dis-satisfied N (%)	Dis-satisfied N (%)	Very dis-satisfied N (%)	Satisfaction score (N)	Very satisfied N (%)	Satisfied N (%)	Neither satisfied nor dis-satisfied N (%)	Dis-satisfied N (%)	Very dis-satisfied N (%)	Satisfaction score (N)
201,601	628 (61.99)	341 (33.66)	28 (2.76)	16 (1.58)	0 (0)	85.36 (1013)	289 (32.51)	367(41.28)	98 (11.02)	135(15.19)	0 (0)	63.70 (889)
201,602	536 (53.44)	433 (43.17)	17 (1.69)	17 (1.69)	0 (0)	**82.78** (1003)	149 (15.68)	549(57.79)	146 (15.37)	106(11.16)	0 (0)	**59.33** (950)
201,603	414 (42.95)	514 (53.32)	16 (1.66)	3 (0.31)	17 (1.76)	86.69 (964)	139 (14.80)	642(68.37)	125 (13.31)	14 (1.49)	19 (2.02)	77.93 (939)
201,604	376 (36.68)	620 (60.49)	18 (1.76)	3 (0.29)	8 (0.78)	86.21 (1025)	102 (10.11)	623(61.74)	253 (25.07)	26 (2.58)	5 (0.50)	75.32(1009)
201,605	699 (69.90)	262 (26.20)	19 (1.90)	6 (0.60)	14 (1.40)	92.18 (1000)	424 (45.30)	312(33.33)	117 (12.50)	37 (3.95)	46 (4.91)	80.65 (936)
201,606	746 (67.09)	294 (26.44)	50 (4.50)	11 (0.99)	11 (0.99)	91.23 (1112)	488 (50.31)	228(23.51)	176 (18.14)	66 (6.80)	12 (1.24)	82.04 (970)
201,607	951 (64.26)	421 (28.45)	79 (5.34)	23 (1.55)	6 (0.41)	90.68 (1480)	538 (41.23)	425(32.57)	199 (15.25)	108 (8.28)	35 (2.68)	78.91(1305)
201,608	725 (67.13)	311 (28.80)	32 (2.96)	10 (0.93)	2 (0.19)	92.22 (1080)	462 (44.17)	439(41.97)	139 (13.29)	5 (0.48)	1 (0.10)	85.05(1046)
201,609	709 (50.32)	590 (41.87)	88 (6.25)	18 (1.28)	4 (0.28)	87.95 (1409)	405 (31.35)	571(44.20)	307 (23.76)	9 (0.70)	0 (0)	81.17(1292)
201,610	843 (74.87)	194 (17.23)	23 (2.04)	56 (4.97)	10 (0.89)	91.37 (1126)	481 (47.72)	351(34.82)	125 (12.40)	46 (4.56)	5 (0.50)	84.38(1008)
201,611	766 (67.61)	253 (22.33)	89 (7.86)	17 (1.50)	8 (0.71)	90.63 (1133)	426 (43.20)	303(30.73)	186 (18.86)	61 (6.19)	10 (1.01)	80.96 (986)
201,612	714 (67.04)	126 (11.83)	214 (20.09)	7 (0.66)	4 (0.38)	88.76 (1065)	475 (51.57)	255(27.69)	136 (14.77)	47 (5.10)	8 (0.87)	84.12 (921)
201,701	719 (76.82)	28 (2.99)	171 (18.27)	18 (1.92)	0 (0)	90.75 (936)	540 (61.57)	135(15.39)	177 (20.18)	25 (2.85)	0 (0)	86.85 (877)
201,702	726 (73.63)	213 (21.60)	39 (3.96)	5 (0.51)	3 (0.30)	**93.44** (986)	698 (74.65)	89 (9.52)	86 (9.20)	54 (5.78)	8 (0.86)	**89.52** (935)

Bold signified maximum and minimum satisfaction scores during January 2016-Febraury 2017

## Abbreviations

APP: Application
ATM: Automatic Teller Machine
LED: Light Emitting Diode
UK: United Kingdom
US: United States
WHO: World Health Organization
